# Combined Vitamin D, Omega-3 Fatty Acids, and a Simple Home Exercise Program May Reduce Cancer Risk Among Active Adults Aged 70 and Older: A Randomized Clinical Trial

**DOI:** 10.3389/fragi.2022.852643

**Published:** 2022-04-25

**Authors:** Heike A. Bischoff-Ferrari, Walter C. Willett, JoAnn E. Manson, Bess Dawson-Hughes, Markus G. Manz, Robert Theiler, Kilian Braendle, Bruno Vellas, René Rizzoli, Reto W. Kressig, Hannes B. Staehelin, José A. P. Da Silva, Gabriele Armbrecht, Andreas Egli, John A. Kanis, Endel J. Orav, Stephanie Gaengler

**Affiliations:** ^1^ Center on Aging and Mobility, University Hospital Zurich, Zurich City Hospital-Waid and University of Zurich, Zurich, Switzerland; ^2^ Department of Aging Medicine and Aging Research, University Hospital Zurich and University of Zurich, Zurich, Switzerland; ^3^ University Clinic for Aging Medicine, Zurich City Hospital-Waid, Zurich, Switzerland; ^4^ Department of Epidemiology and Department of Nutrition, Harvard T. H. Chan School of Public Health, Boston, MA, United States; ^5^ Department of Medicine, Brigham and Women’s Hospital, Harvard Medical School, Boston, MA, United States; ^6^ Jean Mayer USDA Human Nutrition Research Center on Aging, Tufts University, Boston, MA, United States; ^7^ Department of Medical Oncology and Hematology, University Hospital Zurich, University of Zurich, Zurich, Switzerland; ^8^ UMR INSERM 1027, Gérontopôle de Toulouse, Institut Du Vieillissement, Center Hospitalo-Universitaire de Toulouse, Toulouse, France; ^9^ Division of Bone Diseases, Faculty of Medicine, Geneva University Hospitals, Geneva, Switzerland; ^10^ University Department of Geriatric Medicine Felix Platter, University of Basel, Basel, Switzerland; ^11^ Centro Hospitalar e Universitário de Coimbra, Coimbra Institute for Clinical and Biomedical Research (iCBR), Faculty of Medicine, University of Coimbra, Coimbra, Portugal; ^12^ Klinik für Radiologie, Charité-Universitätsmedizin Berlin, Corporate Member of Freie Universität Berlin and Humboldt-Universität zu Berlin, Berlin, Germany; ^13^ Center for Metabolic Diseases, University of Sheffield Medical School, Sheffield, United Kingdom; ^14^ Mary MacKillop Institute for Health Research, Australian Catholic University, Melbourne, VIC, Australia; ^15^ Department of Biostatistics, Harvard T. H. Chan School of Public Health, Boston, MA, United States

**Keywords:** vitamin D, omega-3, exercise, prevention, cancer, healthy aging, co supplementation, combined treatment

## Abstract

**Objective:** The aim of this study was to test the individual and combined benefit of vitamin D, omega-3, and a simple home strength exercise program on the risk of any invasive cancer.

**Design:** The DO-HEALTH trial is a three-year, multicenter, 2 × 2 × 2 factorial design double-blind, randomized-controlled trial to test the individual and combined benefit of three public health interventions.

**Setting:** The trial was conducted between December 2012 and December 2017 in five European countries.

**Participants:** Generally healthy community-dwelling adults ≥70 years were recruited.

**Interventions:** Supplemental 2000 IU/day of vitamin D_3_, and/or 1 g/day of marine omega-3s, and/or a simple home strength exercise (SHEP) programme compared to placebo and control exercise.

**Main outcome:** In this pre-defined exploratory analysis, time-to-development of any verified invasive cancer was the primary outcome in an adjusted, intent-to-treat analysis.

**Results:** In total, 2,157 participants (mean age 74.9 years; 61.7% women; 40.7% with 25-OH vitamin D below 20 /ml, 83% at least moderately physically active) were randomized. Over a median follow-up of 2.99 years, 81 invasive cancer cases were diagnosed and verified. For the three individual treatments, the adjusted hazard ratios (HRs, 95% CI, cases intervention versus control) were 0.76 (0.49–1.18; 36 vs. 45) for vitamin D_3_, 0.70 (0.44–1.09, 32 vs. 49) for omega-3s, and 0.74 (0.48–1.15, 35 vs. 46) for SHEP. For combinations of two treatments, adjusted HRs were 0.53 (0.28–1.00; 15 vs. 28 cases) for omega-3s plus vitamin D_3_; 0.56 (0.30–1.04; 11 vs. 21) for vitamin D_3_ plus SHEP; and 0.52 (0.28–0.97; 12 vs. 26 cases) for omega-3s plus SHEP. For all three treatments combined, the adjusted HR was 0.39 (0.18–0.85; 4 vs. 12 cases).

**Conclusion:** Supplementation with daily high-dose vitamin D_3_ plus omega-3s, combined with SHEP, showed cumulative reduction in the cancer risk in generally healthy and active and largely vitamin D–replete adults ≥70 years.

**Clinical Trial Registration:** ClinicalTrials.gov, Identifier: NCT01745263.

## Introduction

As the incidence of most cancers increases markedly with age, and cancer is the second leading cause of mortality in older adults, cancer is considered a major age-related disease in the United States and Europe (White et al., 2014; Laconi et al., 2020). Furthermore, accelerated aging and cancer development appear to be promoted by some of the same lifestyle-risk factors, such as low physical activity and an unhealthy diet (Lopez-Otin et al., 2013).

However, apart from some preventive recommendations such as smoking cessation for lung cancer (Dragnev et al., 2003), public health efforts that focus on cancer prevention at midlife and older age have been largely focused on vaccination and screening efforts (White et al., 2014; Emmons and Colditz, 2017). This may in part be explained by mixed findings from clinical trials that tested single public health interventions for cancer prevention (White et al., 2014). Alternatively, combined interventions taking advantage of potentially small additive benefits from several public health strategies are largely lacking (Sabia et al., 2012). Although novel cancer treatments aim to block multiple pathways for cancer development by combining several agents (Bayat Mokhtari et al., 2017), this concept has not been translated into cancer prevention (Sabia et al., 2012).

With regard to vitamin D and cancer prevention, in mechanistic studies, vitamin D inhibits the growth of cancer cells by regulating several genes responsible for cell proliferation and differentiation (James et al., 1996; Kawa et al., 1996; Campbell et al., 1997; Yanagisawa et al., 1999; Diaz et al., 2000; Sundaram et al., 2000; Gaschott and Stein, 2003; Jiang et al., 2004; Li et al., 2004; Kumagai et al., 2005; Moreno et al., 2005). Furthermore, observational studies support an inverse association between vitamin D blood levels and total cancer risk (Han et al., 2019). However, data from clinical trials testing supplemental vitamin D are mixed, with an overall suggestion that vitamin D has no benefit on cancer prevention (Haykal et al., 2019; Keum et al., 2019; Chandler et al., 2020) but may reduce the risk of advanced cancer and fatal cancer (Haykal et al., 2019; Keum et al., 2019; Chandler et al., 2020).

With regard to omega-3 and cancer prevention, mechanistic studies show that omega-3s may inhibit carcinogenesis (Larsson et al., 2004) by suppression of inflammation, cell proliferation, and angiogenesis (Fay et al., 1997; Larsson et al., 2004; Calviello et al., 2007). In addition, the importance of lipid metabolism of cancer cells has gained the attention of cancer research more recently, which led to the proposal of another mechanism by which both omega-3 and omega-6 supplements act on cancer cell death of acidic cancer cells by ferroptosis (Dierge et al., 2021). However, cohort studies on the association of omega-3 and total cancer risk have been inconclusive (MacLean et al., 2006). In addition, a 2020 meta-analysis of 27 trials with 113,557 participants treated with a mean dose of 1.7 g of omega-3 over a mean duration of 32 months (Hanson et al., 2020) suggested little or no benefit on the risk of any cancer diagnosis or cancer death (Hanson et al., 2020).

Exercise may reduce cancer risk by several mechanistic pathways including a decrease in inflammation and improved immune function (McTiernan, 2008; Hong and Lee, 2020; Wang and Zhou, 2020). Consistently, observational studies support that higher physical activity reduces risk of several cancers (Rezende et al., 2018) and increases cancer survival (Cormie et al., 2017). However, clinical trials that tested the effect of exercise on cancer prevention are still lacking (McTiernan et al., 1999).

The aim of the present exploratory trial among 2,157 generally healthy adults aged 70 and older was to address these knowledge gaps by testing the effect of daily high-dose vitamin D_3_, daily supplemental omega-3s, and a simple home exercise program (SHEP), alone and in combination, on the risk of any invasive cancer among adults aged 70 and older.

## Methods

### Trial Design

The DO-HEALTH trial is a multicenter, double-blind, and 2 × 2 × 2 factorial design randomized-controlled clinical trial designed to support healthy aging in European adults aged 70 years and older (NCT01745263) addressing six primary endpoints (Bischoff-Ferrari et al., 2020). A verified cancer risk was a pre-defined exploratory outcome including all 2,157 participants. Clinical visits were at baseline, one, two, and three years, and there were phone calls every 3 months. The detailed trial design and protocol are provided elsewhere (Bischoff-Ferrari et al., 2021).

### Participants

We targeted 2,157 participants who were generally healthy community-dwelling adults and recruited them from seven centers in five European countries, namely, Switzerland, Germany, Austria, France, and Portugal (Bischoff-Ferrari et al., 2021). The inclusion criteria were the absence of major health events in the 5 years prior to enrollment including cancer diagnosis, recurrence, treatment, sufficient mobility to come to the study centers, and good cognitive function with an MMSE score of at least 24 (Bischoff-Ferrari et al., 2020). The participants were required to limit the use of vitamin D from all supplemental sources to the recommended dietary allowance intake for older adults (800 IU per day), and to forego any supplemental omega-3 intake (Bischoff-Ferrari et al., 2021). All the participants signed the informed consent, and the ethical and regulatory agencies of all five countries approved the study protocol.

### Randomization and Masking

We randomized participants to eight treatment groups using block randomization (block sizes of 16 individuals): 2000 IU/day of vitamin D_3_ and 1 g/day of omega-3s and SHEP (*n* = 264); vitamin D_3_ and marine omega-3s (*n* = 265); vitamin D_3_ and SHEP (*n* = 275); vitamin D_3_ alone (*n* = 272); omega-3s and SHEP (*n* = 275); omega-3s alone (*n* = 269); SHEP alone (*n* = 267); or placebo (*n* = 270). Each participant received two study capsules per day identical in size, appearance, taste, and weight, and all capsules had coatings to prevent unblinding by the aftertaste. Each active omega-3s capsule contained 500 mg of eicosapentaenoic acid (EPA) and docosahexaenoic acid (DHA) in a ratio of 1:2; each active vitamin D capsule contained 1000 IU of Vitamin D_3_ stabilized with dl-α-tocopherol (vitamin E, 2.5 pro mill); and each placebo capsule contained high-oleic sunflower oil. The SHEP and control exercise program are outlined in Supplementary Table S1 in the supplement. The dosing of the intervention was based on the main outcomes of the trial, including cardiovascular health, bone health, muscle health, brain health, and immunity. Details on the evidence in the literature can be found in Bischoff-Ferrari et al 2021 (Bischoff-Ferrari et al., 2021). The consort flow diagram was published elsewhere (Bischoff-Ferrari et al., 2020).

### Cancer Outcomes

The risk of verified invasive cancer was a pre-defined exploratory analysis of the DO-HEALTH trial, with time to any verified invasive cancer as the primary outcome. The pre-defined **s**econdary endpoints were the prevention of three site-specific cancers, namely, gastro-intestinal cancer, prostate cancer in men, and breast cancer in women (Bischoff-Ferrari et al., 2021). Cancer events were assessed prospectively at each in-person contact every 3 months throughout the 3-year follow-up, in all 2,157 participants. All reported new cancer events triggered a detailed report on location, duration, and treatment of the cancer and were documented by the medical record review to verify the reported new cancer event. The medical reviews of the reported cancer events were carried out by an Independent Physician Endpoint Committee. We considered all cancer events, including ICD-10 D03:D04, without non-melanoma skin cancer (ICD-10: C44) and benign neoplasms (ICD-10: D13). The earliest reported date of the verified event was used as the outcome time point, with censoring at death (*n* = 25) (Bischoff-Ferrari et al., 2020) or at the end of follow-up.

The history of cancer prior to the 5-year study eligibility window was assessed using the medical history recorded by the study physician at the baseline visit, documenting cancer onset and ICD-10 codes (Bischoff-Ferrari et al., 2021).

### Statistical Analysis

We analyzed the trial based on the intent-to-treat. For the primary analysis, we included only the 81 participants whose self-reported invasive cancer could be verified as cases. To assess the effect of treatment on the incidence of any invasive cancer, we fitted Cox-proportional hazard models, adjusted for history of cancer, sex, body mass index (BMI), prior fall, study site, and age (70–84 and 85+) (Bischoff-Ferrari et al., 2020). The primary predictors were indicator variables for the three treatments since we did not find significant interactions between the treatments in preliminary testing. We evaluated combination treatments within subgroups of participants, comparing participants who had a particular combination of treatments to participants who had neither/none of the treatments. For example, participants who were randomized to both Vitamin D_3_ and omega-3s (regardless of exercise status) were compared to participants who were randomized to neither Vitamin D_3_ nor omega-3s (regardless of exercise status). Because of the factorial design, about half of the participants in each of these two groups received the SHEP intervention. Sensitivity analyses excluded participants with any history of invasive cancer or cases from participants who self-reported invasive cancer, even if they were not verified. Regarding the small numbers of cases of site-specific cancers and cancer deaths, we used the same statistical approach but presented as unadjusted model results.

Given that our statistical models report on several comparisons, we caution on the interpretation of *p*-values (Di Leo and Sardanelli, 2020). Following the recent warnings by the American Statistical Association regarding this issue, we do not classify results as statistically significant or not based on a *p*-value threshold (Di Leo and Sardanelli, 2020). Instead, we consider results with *p*-values < 0.05 as evidence for rejecting the null hypothesis. We also examine the magnitudes of the estimates and the absolute number of cancer cases for each comparison (Di Leo and Sardanelli, 2020). To provide an estimate for clinical relevance, we calculated the probabilities of remaining cancer-free cases at 3 years from Kaplan–Meier curve analysis and, from those, the number needed to treat (NNT) in order to prevent one incident case of cancer at 3 years follow-up (Altman and Andersen, 1999). Statistical analyses were performed in SAS (v9.4) and R (v. 4.02) within Rstudio (v. 1v2.1578).

### Patient and Public Involvement

The patients and the public were not involved in setting up the research question, design, outcome measures, interpretation of the results, or writing the manuscript.

## Results

### Trial Participants

Table 1 shows the baseline characteristics of all 2,157 participants. Overall, the characteristics of the treatment and non-treatment groups were balanced. The mean age was 74.9 years, 61.7% were women, BMI 26.3 (SD 4.3) (kg/m^2^); and only 5.2% were active smokers. The participants reported on average 3.3 comorbidities (SD: 3.0), had good mobility [median SPPB score: 11.0 out of 12 (IQR: 10.0–12.0)], and the large majority of participants (82.6%) were physically active at the baseline engaging in moderate-to-high physical activity levels and vitamin D replete (59.3%). Overall, 24% of participants were taking vitamin D supplements at the baseline, with 33.0% taking vitamin D supplements up to 800 IU vitamin D per day in addition to the study medication at year three.

**TABLE 1:** Baseline characteristics of the DO-HEALTH trial participants.

	Vitamin D		*p*	Omega-3		*p*	Exercise		*p*	Overall
	Vitamin D	No vitamin D		Omega-3	No Omega-3		SHEP	Control Exercise		Total
	(*n* = 1,076)	(*n* = 1,081)		(*n* = 1,073)	(*n* = 1,084)		(*n* = 1,081)	(*n* = 1,076)		(*n* = 2,157)
Age, years, mean (SD)	75.0 (4.5)	74.9 (4.4)	0.50	74.7 (4.3)	75.2 (4.6)	0.02	75.0 (4.5)	74.9 (4.4)	0.72	74.9 (4.4)
Age categories, n (%)	—	—	0.52	—	—	0.08	—	—	0.89	—
70–74 years	606 (56.3)	631 (58.4)	—	635 (59.2)	602 (55.5)	—	622 (57.5)	615 (57.2)	—	1,237 (57.3)
75–84 years	417 (38.8)	405 (37.5)	—	398 (37.1)	424 (39.1)	—	408 (37.7)	414 (38.5)	—	822 (38.1)
85 + years	53 (4.9)	45 (4.2)	—	40 (3.7)	58 (5.4)	—	51 (4.7)	47 (4.4)	—	98 (4.5)
BMI, kg/m^2^, mean (SD)	26.5 (4.4)	26.2 (4.2)	0.08	26.3 (4.2)	26.4 (4.3)	0.72	26.3 (4.2)	26.4 (4.4)	0.57	26.3 (4.3)
Sex, no. (%)	—	—	0.79	—	—	0.60	—	—	0.86	—
Women, *n* (%)	667 (62.0)	664 (61.4)		668 (62.3)	663 (61.2)	—	665 (61.5)	666 (61.9)		1,331 (61.7)
Men, *n* (%)	409 (38.0)	417 (38.6)		405 (37.7)	421 (38.8)	—	416 (38.5)	410 (38.1)		826 (38.3)
Education, years, mean (SD)	12.7 (4.4)	12.6 (4.2)	0.73	12.6 (4.2)	12.7 (4.4)	0.66	12.6 (4.2)	12.6 (4.4)	0.92	12.6 (4.3)
Comorbidity score, mean (SD)	3.3 (3.1)	3.3 (3.0)	0.71	3.3 (3.1)	3.3 (2.9)	0.92	3.2 (3.0)	3.4 (3.1)	0.20	3.3 (3.0)
Short physical performance battery score, median (IQR)	12.0 (10.0–12.0)	11.0 (10.0–12.0)	0.28	11.0 (10.0–12.0)	11.0 (10.0–12.0)	0.90	11.0 (10.0–12.0)	11.0 (10.0–12.0)	0.67	11.0 (10.0–12.0)
Vitamin D supplement users (≥800 IU), *n* (%)	110 (10.2)	126 (11.7)	0.29	123 (11.5)	113 (10.4)	0.44	127 (11.7)	109 (10.1)	0.23	236 (10.9)
Vitamin D insufficiency (<20 ng/ml), *n* (%)	427 (40.1)	445 (41.4)	0.52	422 (39.7)	450 (41.8)	0.33	422 (39.4)	450 (42.1)	0.20	872 (40.7)
Serum vitamin D concentration, ng/mL, mean (SD)	22.4 (8.4)	22.4 (8.5)	0.85	22.4 (8.4)	22.4 (8.4)	0.98	22.8 (8.6)	22.0 (8.2)	0.03	22.4 (8.4)
Serum DHA concentration, µg/mL, mean (SD)	78.1 (37.9)	78.1 (35.9)	0.97	78.9 (37.2)	77.3 (36.6)	0.32	78.2 (36.5)	78.0 (37.4)	0.93	78.1 (36.9)
Serum EPA concentration, µg/mL, median (IQR)	24.8 (17.4–37.7)	26.2 (18.6–37.7)	0.14	26.1 (18.5–37.7)	25.3 (17.6–37.9)	0.56	25.1 (17.5–37.6)	25.9 (18.6–38.1)	0.24	25.5 (18.1–37.7)
Serum omega-3 PUFA concentration, *n* (%)	—	—	0.42	—	—	0.91	—	—	0.95	—
<100 μg/ml	541 (50.9)	527 (49.1)	—	528 (49.9)	540 (50.1)	—	535 (50.1)	533 (49.9)	—	1,068 (50.0)
≥100 μg/ml	523 (49.1)	546 (50.9)		531 (50.1)	538 (49.9)		534 (50.0)	535 (50.1)		1,069 (50.0)
Physical activity level, *n* (%)	—	—	0.08	—	—	0.47	—	—	0.47	—
None	207 (19.3)	168 (15.6)	—	190 (17.7)	185 (17.1)	—	179 (16.6)	196 (18.2)	—	375 (17.4)
1–2 times per week	318 (29.6)	334 (30.9)	—	311 (29.0)	341 (31.5)	—	323 (29.9)	329 (30.6)	—	652 (30.3)
≥3 times per week	550 (51.2)	578 (53.5)	—	570 (53.2)	558 (51.5)	—	578 (53.5)	550 (51.2)	—	1,128 (52.3)

Differences between treated and non-treated participants at the baseline were tested using the Wilcoxon rank sum test, t-test, or chi-square test, for non-normal, normal, and categorical variables, respectively. Medians and IQRs are presented for variables with skewness >1.5. Percentages are rounded to 1 decimal, which could lead to percentage sums of 100.1% or 99.9%. The body mass index (BMI) was calculated as weight in kilograms divided by height in meters squared. Higher BMI, values reflect overweight (≥25) and obesity (≥30).

### Any Verified Invasive Cancer

**FIGURE 1:**
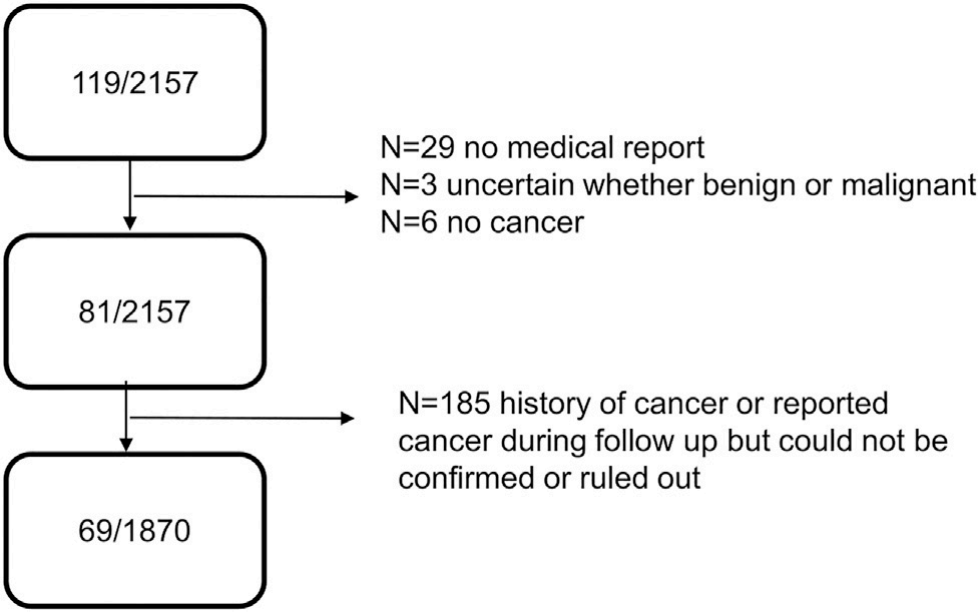
Flow chart cancer cases DO-HEALTH.

Among 2,157 participants and 5,562.4 person-years of follow-up (median 2.99 years), 119 invasive cancer events were self-reported during the in-person interviews every 3 months (Figure 1). Of those, 29 could not be verified with a medical report, and for three cases, the independent physician committee could not decide if the neoplasm was benign or malignant. Furthermore, for six reported cancer cases, a medical report verified a noncancerous nature of the neoplasm. This left 81 cases with verified invasive cancer for the main intent-to-treat analysis.

Given that there was no significant effect modification of the treatment effects by pre-defined subgroups, we did not proceed with subgroup analyses (Supplementary Table S2).

### Intent-to-Treat Analysis

**FIGURE 2:**
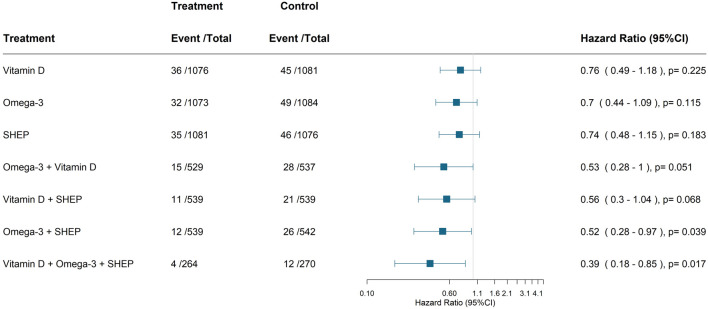
Primary endpoint—effect of treatments on the prevention of any invasive cancer. Cox-proportional hazard model adjusted for history of cancer, sex, BMI, prior fall, age, and study center. The comparison group is always the group that does not have the respective treatment(s) of interest. For all three treatments, it is the group who received only the placebo. All verified new invasive cancer cases (*n* = 81) among all 2,157 participants. Abbreviation: SHEP, Simple home exercise program.

Including all 2,157 participants and 81 verified new invasive cancer cases, Figure 2 displays the individual and combined benefits of the treatments.

For the three individual treatments, adjusted hazard ratios (HRs, 95% CI, cases intervention versus control) were 0.76 (0.49–1.18; 36 vs. 45) for vitamin D_3_; 0.70 (0.44–1.09, 32 vs. 49) for omega-3s; and 0.74 (0.48–1.15, 35 vs. 46) for SHEP. For combinations of two treatments, adjusted hazard ratios were 0.53 (0.28–1.00; 15 vs. 28 cases) for omega-3s plus vitamin D_3_; 0.56 (0.30–1.04; 11 vs. 21) for vitamin D_3_ plus SHEP; and 0.52 (0.28–0.97; 12 vs. 26 cases) for omega-3s plus SHEP. For all three treatments combined, the adjusted hazard ratio was 0.39 (0.18–0.85; 4 vs. 12 cases).

The NNT in order to prevent one incident case of cancer at the 3-year follow-up with the three treatments combined was 35 (95% CI, 26–137).

### Sensitivity Analyses

**FIGURE 3:**
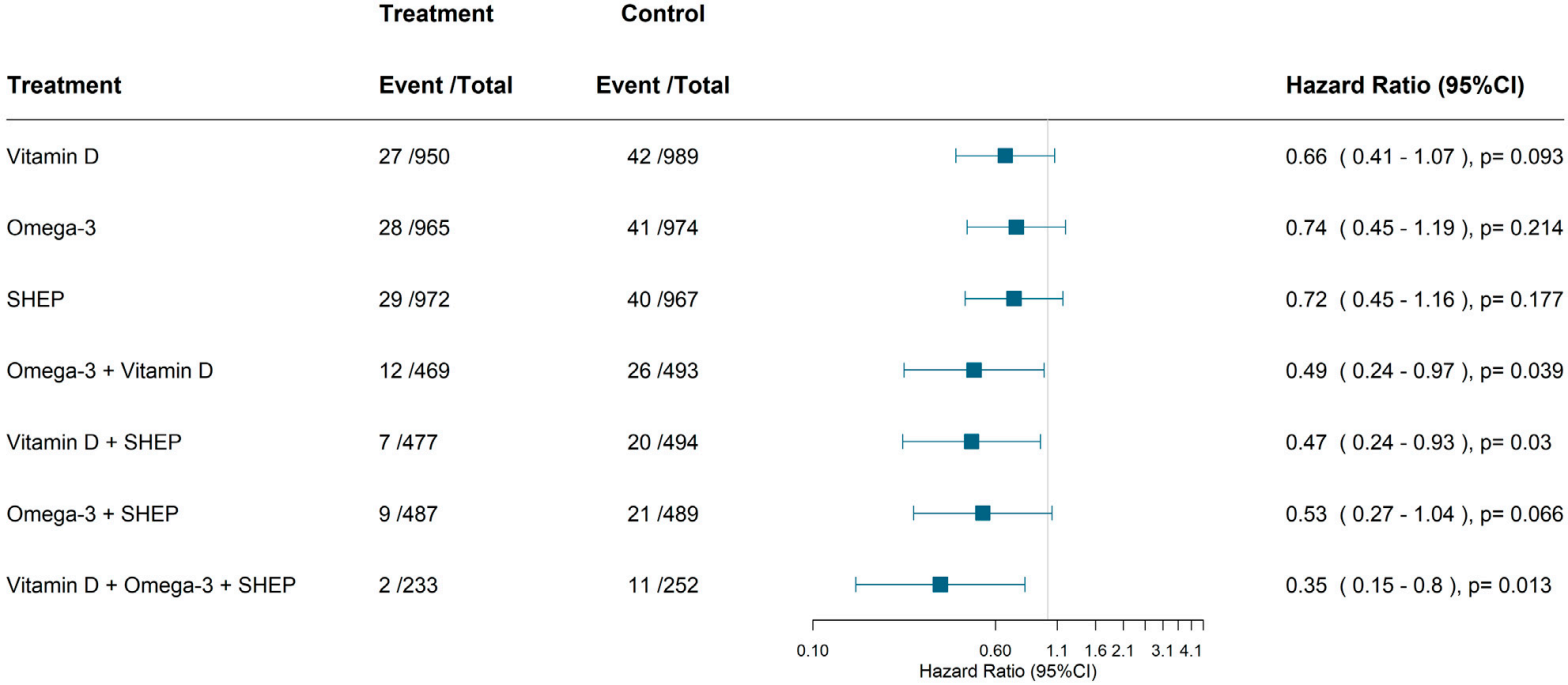
Primary endpoint—sensitivity Analysis—excluding participants with a history of cancer. Cox-proportional hazard model adjusted for sex, BMI, prior fall, age, and study center. The comparison group is always the group that does not have the respective treatment(s) of interested. For all three treatments, it is the group who received only the placebo. All verified new invasive cancer cases (*n* = 69) among 1972 participants without a cancer history. Abbreviation: SHEP, Simple home exercise program.

Excluding 185 participants with a reported history of cancer and a total of 69 verified new invasive cancer cases, Figure 3 displays the individual and combined benefits of the treatments for primary prevention. All three treatments combined resulted in an adjusted hazard ratio of 0.35 (0.15–0.80; 2 vs. 11 cases) and an NNT of 33 (25–108).

Including all 2,157 participants and 113 reported invasive cancer cases (119 minus six verified non-cases), all three treatments combined resulted in an adjusted hazard ratio of 0.51 (0.27–0.98; 9 vs. 16 cases; Supplementary Figure S1).

### Site-specific Cancers and Cancer Mortality

With regard to predefined site-specific cancers (Table 2), there were very low numbers and results must be interpreted with caution. None of the treatments individually or in combination reduced gastrointestinal cancers (*n* = 22) or breast cancer in women (*n* = 13). However, there was a suggestion that omega-3 alone [HR 95% CI = 0.17 (0.04–0.75); 2 vs. 12 cases] and its combination with SHEP [HR 95% CI = 0.12 (0.02–0.74); 0 vs. 6 cases] may reduce prostate cancer risk (*n* = 14). Cancer mortality was not a predefined endpoint, and there were only eight cases (Supplementary Figure S1).

**TABLE 2:** Secondary endpoints: individual cancers.

	Treatment	Events/total	Control	Hazard ratio (95% CI)
		treatment		
Gastro-intestinal cancer^a^	Vitamin D	11/1,076	11/1,081	1.00 (0.43–2.3)
	Omega-3	8/1,073	14/1,084	0.59 (0.25–1.40)
	SHEP	12/1,081	10/1,076	1.19 (0.51–2.7)
	Omega-3 + Vitamin D	4/529	7/537	0.59 (0.18–1.98)
	Vitamin D + SHEP	5/539	4/539	1.19 (0.37–3.8)
	Omega-3 + SHEP	5/539	7/542	0.70 (0.21–2.3)
	All treatments	1/264	4/270	0.70 (0.16–3.03)
Breast cancer^a^	Vitamin D	6/667	7/664	0.83 (0.28–2.48)
	Omega-3	3/668	10/663	0.31 (0.08–1.12)
	SHEP	6/665	7/666	0.87 (0.29–2.58)
	Omega-3 + Vitamin D	1/330	5/326	0.26 (0.05–1.41)
	Vitamin D + SHEP	2/332	3/331	0.72 (0.16–3.36)
	Omega-3 + SHEP	1/337	5/335	0.27 (0.05–1.42)
	All treatments	0/165	2/165	0.22 (0.03–1.66)
Prostate cancer^a^	Vitamin D	8/409	6/417	1.4 (0.48–4.05)
	^b^Omega-3	2/405	12/421	0.17 (0.04–0.75)
	SHEP	6/416	8/410	0.69 (0.24–1.99)
	Omega-3 + Vitamin D	2/199	6/211	0.24 (0.04–1.46)
	Vitamin D + SHEP	2/207	2/208	0.96 (0.22–4.16)
	^b^Omega-3 + SHEP	0/202	6/207	0.12 (0.02–0.74)
	All treatments	0/99	2/105	0.16 (0.02–1.33)

a Unadjusted.

b Significant at 5% levels.

Cox-proportional hazard models are unadjusted due to the small numbers of cancer cases for the site-specific cancers.

## Discussion

In this multicenter clinical trial among generally healthy and active older adults at their peak of age-related cancer risk, high-dose daily vitamin D_3_, supplemental marine omega-3s, and a simple home exercise program had cumulative benefits on the risk of any invasive cancer. Notably, in this predefined exploratory analysis of the DO-HEALTH trial, we saw a cancer risk reduction when all three interventions were combined. This benefit was consistent in sensitivity analyses that excluded adults with a history of cancer, reflecting primary prevention, and when also unverified reported cancer cases were included.

Assessing the effect of both the individual and combined benefit of interventions in the prevention of cancer in a large clinical trial is a rather novel approach. To the best of our knowledge, there are only few very small trials with less than 100 participants that tested the combined benefit of vitamin D and omega-3 in cancer patients. One randomized placebo controlled trial among 81 patients with stage II and III colorectal cancer tested the supplementation of weekly Vitamin D, daily omega-3 supplementation, and their combination over 8 weeks on inflammatory factors and tumor marker carcinoembryonic antigen (Haidari et al., 2020). Inflammatory markers decreased significantly in the co-supplementation group compared to placebo. In another small randomized-controlled trial on co-supplementation of biweekly Vitamin D and daily omega-3 supplementation in 60 women with polycystic ovary syndrome for 8 weeks, a significant increase in plasma total antioxidant capacity levels and decrease in the high-sensitivity C-reactive protein and malondialdehyde levels were documented (Jamilian et al., 2018). Therefore, the results from the DO-HEALTH trial are timely as they represent the first large trial in the framework of cancer prevention, and our findings are supported by two small trials (Jamilian et al., 2018; Haidari et al., 2020).

In meta-analyses of RCTs for vitamin D (Keum et al., 2019) and omega-3 (Hanson et al., 2020), small benefits on cancer prevention could not be excluded, while trial data on the effect of exercise on cancer prevention are largely missing; mechanistic studies support several protective pathways to cancer prevention for all three interventions. Vitamin D can downregulate growth hormones (Koga et al., 1988; Yanagisawa et al., 1999) and suppress cell proliferation in many cancers (Campbell et al., 1997; Sundaram et al., 2000); omega-3s can inhibit carcinogenesis through anti-inflammatory properties, suppress angiogenesis (Larsson et al., 2004; Calviello et al., 2007) or exert ferroptosis of acidic cancer cells through lipid peroxidation (Dierge et al., 2021); and exercise can induce the apoptosis of tumor cells (Wang and Zhou, 2020). Notably, with the 2 × 2 × 2 factorial design of our trial, we may have been able to detect the cumulative benefits of these in part by complementary mechanistic pathways for cancer prevention.

For vitamin D and cancer prevention, our trial tested a daily dose of 2000 IU vitamin D_3_, which according to a recent meta-analysis on cancer prevention, may have reduced cancer death on its own (Keum et al., 2019), while an effect of Vitamin D on cancer incidence was not seen, neither in the 5-year Finnish Vitamin D Trial, where 1600 IU/d and 3200 IU/d were tested against placebo in 2,495 participants ≥60 years (Virtanen et al., 2022), and the meta-analysis by Keum et al. This meta-analysis evaluated ten RCTs (6,537 cases) and included the large VITAL trial (Manson et al., 2019b). Four of the included trials provided a large intermittent bolus of vitamin D_3_ (20,000 IU/week–500,000 IU/year), and six trials provided vitamin D_3_ in a daily dose (400–2000 IU/day) (Keum et al., 2019). Overall, the authors suggested that there was no evidence for the benefit for prevention, but there was for cancer death, and that daily dosing is superior to the bolus dosing of vitamin D (Keum et al., 2019). This pattern showed that bolus dosing of vitamin D may be less effective and is consistent with data from fall and fracture prevention (Smith et al., 2007; Sanders et al., 2010; Bischoff-Ferrari et al., 2016; Ginde et al., 2017; Bolland et al., 2018) and acute respiratory infections (Jolliffe et al., 2020).

Regarding the comparison of our findings with the U.S. VITAL study, our trial utilized the same dose of supplemental omega-3 (1 g/day) (Manson et al., 2019a; Manson et al., 2019b), but there were differences in the composition of omega-3 PUFAs. The supplemental marine omega-3s used in this trial were algae-based (EPA + DHA ratio: 1:2), whereas supplements in VITAL were fish oil–based (EPA + DHA, ratio 1.3:1). In addition, VITAL recruited younger participants (mean age 67.1 years) than those recruited by DO-HEALTH (mean age 74.9 years). Nonetheless, the 95% CIs on the individual effects of both, supplemental omega-3 [VITAL HR (95% CI) = 1.03 (0.93–1.13); DO-HEALTH HR (95% CI) = 0.70 (0.44–1.09)], and vitamin D_3_ [VITAL HR (95%CI) = 0.96 (0.88–1.06); DO-HEALTH HR (95%CI) = 0.76 (0.49–1.18)] overlapped between the two trials regarding the prevention of invasive cancer. However, in DO-HEALTH, there was a cumulative benefit of omega-3s with vitamin D_3_, both in the intent-to-treat analysis [HR (95%CI) = 0.53 (0.28–1.00)] and in the analysis excluding participants with a history of cancer [HR (95%CI) = 0.49 (0.24–0.97)].

Although a recent meta-analysis on randomized-controlled trials could not support a protective effect of omega-3 fatty acids on the prevention of cancer (Hanson et al., 2020), recent animal studies provide a potential mechanism of omega-3 and omega-6 fatty acids on acidic cancer cell death, and therefore affecting the progression of cancer (Dierge et al., 2021). Lipid metabolism may play an important role in cancer cell growth, based on tumor models and animal studies (Snaebjornsson et al., 2020). Compared to mono-unsaturated fatty acids, long-chain polyunsaturated fatty acids lead to reduction in tumor growth through lipid peroxidation and subsequent ferroptosis. This effect increased with the number of double bonds available in fatty acids (Dierge et al., 2021).

To date, DO-HEALTH is the first trial to examine the effect of exercise on cancer prevention. Our results suggest that the simple strength home exercise program may effectively contribute to cancer prevention in combination with supplemental omega-3s and also in combination with both supplemental omega-3s and vitamin D_3_. The potential benefit of exercise on cancer prevention is supported by mechanistic studies (McTiernan, 2008; Hong and Lee, 2020; Wang and Zhou, 2020) and large observational studies (Rezende et al., 2018).

For individual cancers, our numbers were too low for reliable interpretation. Thus, the finding that omega-3 alone or in combination with SHEP may reduce prostate cancer needs to be interpreted with great caution. In VITAL, omega-3 and vitamin D did not reduce individual cancers (breast, gastro-intestinal, and prostate) with a suggestion that omega-3s may slightly increase the risk of prostate cancer [HR (95% CI) = 1.15 (0.94–1.39)] (Manson et al., 2019a). The latter finding is consistent with a recent meta-analysis of seven trials, including the VITAL trial [RR (95% CI) = 1.10 (0.97–1.24)] (Hanson et al., 2020). Notably, however, the potentially harmful effect of omega-3 on prostate cancer was inconsistent in the same meta-analysis because several trials suggested a reduction in PSA levels with supplemental omega-3s (Hanson et al., 2020).

This trial has many strengths, including its prospective assessment of new cancer cases by in-person contacts every 3 months and verification by medical records. Furthermore, adherence to the interventions was high, with on average above 80% for the study medication and on average over 60% for performing the exercise program at least twice a week (Bischoff-Ferrari et al., 2020). Furthermore, the rate of any new invasive cancer in DO-HEALTH participants aged 70 and older was representative for the age-group investigated (White et al., 2014), between 3.8% (81 verified cases) and 5.2% (113 reported cases), compared with 1.2% in VITAL participants aged 50 years and older and a little less compared to the Finnish Vitamin D Trial with 5.2% (129 recorded cases) in participants aged ≥60 years (Virtanen et al., 2022). Finally, the consistency of findings between the primary analysis and sensitivity analyses lends strength to our findings. In addition, DO-HEALTH was designed as a complementary European trial to VITAL, which addressed cancer as a primary endpoint (Manson et al., 2019a; Manson et al., 2019b).

There are also limitations. First, any new invasive cancer was a pre-defined exploratory outcome and with 2,157 participants, the size of our trial may be considered small for a cancer endpoint trial, which explains the definition of cancer as an exploratory endpoint in DO-HEALTH (Bischoff-Ferrari et al., 2021). Furthermore, for cancer prevention, the duration of our trial would be considered short. In general, many years may be needed to see the effects of decreasing exposures involved in the early stages of carcinogenesis, such as smoking (Reid et al., 2006). On the other hand, reduction in risk factors that act late in carcinogenesis may result in rapid decreases in cancer risk (Kim and Giovannucci, 2020); for example, the cessation of hormone therapy and risk of breast cancer (Collaborative Group on Hormonal Factors in Breast, 2019), or vitamin D supplementation and advanced cancer (Haykal et al., 2019; Keum et al., 2019; Chandler et al., 2020; Kim and Giovannucci, 2020).

In conclusion, this is the first randomized-controlled trial to investigate the combination of three complementary treatments for the prevention of cancer and suggest that the combination of daily vitamin D_3_, supplemental marine omega-3s, and a simple home exercise program may be effective in the prevention of invasive cancer among generally healthy and active adults aged 70 and older. Based on our findings, with the triple combination, we would need to treat 35 persons (95% CI, 26–137) in order to prevent one incident case of cancer at 3 years follow-up. These results may shape the future mind-set toward a multicomponent prevention strategy of cancer. Our results, although based on multiple comparisons and requiring replication, may prove to be beneficial for reducing the burden of cancer. Future studies should verify the benefit of combined treatments in the prevention of cancer, also extending to longer follow-ups beyond the 3-year duration assessed in this trial.

## The DO-HEALTH Research Group

The DO-HEALTH Research Group DO-HEALTH Consortium (in bold: Governing Board members; in bold and underlined: Chair; underlined: Team members). **Heike A Bischoff-Ferrari**, DO-HEALTH Coordinator, Principal Investigator and Zurich Site Investigator, leads all endpoints analyses and co-leads the studies “DO-HEALTH health economic model,” “novel biomarkers of immunity,” “novel biomarkers of muscle and bone communication,” Andreas Egli andSandrine Rival, University Hospital Zurich, University of Zurich and Waid and Triemli City Hospital, Zurich, Switzerland; **Bruno Vellas**, Toulouse Site Investigator, contributes to the primary endpoint cognitive decline; Sophie Guyonnet, CHU Toulouse and University of Toulouse III, Toulouse, France; **René Rizzoli**, Geneva Site Investigator, contributes to all bone- and muscle-related endpoints and explores the contribution of protein intake to the benefit of the interventions; Emmanuel Biver and Fanny Merminod RD, Geneva University Hospitals and Faculty of Medicine, Geneva, Switzerland; **Reto W. Kressig**, Basel Site Investigator, contributes to gait analyses and dual task assessments, and Stephanie Bridenbaugh, University Department of Geriatric Medicine FELIX PLATTER and University of Basel, Basel, Switzerland; Norbert Suhm, Department. of Traumatology, University Hospital Basel, contributes to fracture healing study DO-HEALTH; **José A. P. Da Silva**, Coimbra Site Investigator, explores the treatment effects on vertebral fractures, and musculoskeletal pain and function, Centro Hospitalar e Universitário de Coimbra, and Faculty of Medicine, University of Coimbra, Coimbra, Portugal; Cátia C. M. Duarte, Centro Hospitalar e Universitário de Coimbra, Coimbra, Portugal; Ana Filipa Pinto R. N., Faculty of Medicine, University of Coimbra, Coimbra, Portugal; **Dieter Felsenberg**, Berlin Site Investigator, performed the central DO-HEALTH DEXA quality control and evaluation of DEXA measurements; Hendrikje Börst Dipl.Wiss-org, and Gabriele Armbrecht, Charité—Universitätsmedizin Berlin, corporate member of Freie Universität Berlin and Humboldt-Universität zu Berlin, Berlin, Germany; **Michael Blauth**, Innsbruck Site Investigator, explores the functionality after fracture; Anna Spicher, Medical University of Innsbruck, Innsbruck, Austria; **David T. Felson**, co-leads “DO-HEALTH osteoarthritis study,” Manchester Academic Health Science Center, Manchester, United Kingdom and Boston University School of Medicine, Boston, MA, United States; **John A. Kanis**, leads the study “contribution of fall risk to absolute fracture risk within the FRAX model,” University of Sheffield Medical School, Sheffield, United Kingdom and Australian Catholic University, Melbourne, Victoria, Australia; Eugene V. Mccloskey, co-leads the study “contribution of fall risk to absolute fracture risk within the FRAX model,” University of Sheffield, Sheffield, United Kingdom; Helena Johansson, University of Sheffield Medical School, Sheffield, United Kingdom and Catholic University of Australia, Melbourne, Victoria, Australia; **Bernhard Watzl**, co-leads the study “novel biomarkers of immunity,” Manuel Rodriguez Gomez, Max Rubner-Institut, Karlsruhe, Germany; **Lorenz C. Hofbauer**, co-leads the study “novel biomarkers of muscle and bone communication,” Elena Tsourdi and Martina Rauner, Dresden University Medical Center and Center for Regenerative Therapies Dresden, Dresden, Germany; **Uwe Siebert**, co-leads the study “DO-HEALTH health economic model,” UMIT—University for Health Sciences, Medical Informatics and Technology, Hall i.T., Austria and Harvard T. H. Chan School of Public Health, Boston, MA, United States and Massachusetts General Hospital, Harvard Medical School, Boston, MA, United States; **John A. Kanis**, leads DO-HEALTH impact and communication of osteoporosis-related findings on a broad level, and Philippe Halbout PhD, IOF; **Stephen M. Ferrari**, leads DO-HEALTH software development (electronic data capture system and interactive practical software for seniors and healthcare professionals that teaches main findings of DO-HEALTH), Ferrari Data Solutions, Feldmeilen, Switzerland; **Benno Gut**, leads DO-HEALTH visual communication (SHEP avatar) and DO-HEALTH corporate design structures (logo, website software, and communication tools), gut pictures, Horgen, Switzerland; **Marième Ba**, was the DO-HEALTH independent clinical monitoring partner, Pharmalys, Borehamwood, United Kingdom; **Jonas Wittwer Schegg**, industrial partner representative bringing expertise and facilities in plasma analytics for 25-hydroxyvitamin D and omega-3 fatty acids and providing the study medication (vitamin D, omega-3 fatty acids); Stéphane Etheve, DSM Nutritional Products, Kaiseraugst, Switzerland; Manfred Eggersdorfer, University Medical Center Groningen, Gronigen, Netherlands; **Carla Sofia Delannoy**, industrial partner representative providing financial support to DO-HEALTH central coordination, Nestlé Health Science, Lausanne, Switzerland; **Monika Reuschling**, industrial partner representative providing assays for the large DO-HEALTH biomarker study to define reference ranges of common biomarkers in adults aged 70+, Roche diagnostiscs, Rotkreuz, Switzerland.

DO-HEALTH Scientific Advisory Board members and collaborators on specific outcomes **Endel J. Orav**, Harvard T. H. Chan School of Public Health; Boston, MA, United States; **Walter C. Willett**, Harvard T. H. Chan School of Public Health, Boston, MA, United States; **JoAnn E. Manson**, Brigham and Women’s Hospital, Harvard Medical School, Boston, MA, United States; **Bess Dawson-Hughes**, Tufts University, Boston, MA, United States; **Hannes B. Staehelin**, University of Basel, Basel, Switzerland; **Paul W. Walter**, University of Basel, Basel, Switzerland; **Walter Dick**, University of Basel, Basel, Switzerland; **Michael Fried**, University of Zurich, Zurich, Switzerland; **Arnold von Eckardstein**, University of Zurich, Zurich, Switzerland; **Robert Theiler**, University Hospital Zurich and University of Zurich, Zurich, Switzerland; **Hans-Peter Simmen**, University of Zurich, Zurich, Switzerland; **Wolfgang Langhans**, ETH Zurich, Zurich, Switzerland; **Annelies Zinkernagel**, University Hospital of Zurich, Zurich, Switzerland; **Nicolas Mueller**, University Hospital of Zurich, Zurich, Switzerland; **Oliver Distler**, University Hospital of Zurich, Zurich, Switzerland; **Klaus Graetz**, University Hospital of Zurich, Zurich, Switzerland; **Ina Nitschke**, University Hospital of Zurich, Zurich, Switzerland; **Thomas Dietrich**, University of Birmingham, United Kingdom; **Walter Baer**, University of Zurich, Zurich, Switzerland; **Klara Landau**, University Hospital of Zurich, Zurich, Switzerland; **Frank Ruschitzka**, University Hospital of Zurich, Zurich, Switzerland; **Markus Manz**, University Hospital of Zurich, Zurich, Switzerland; **Peter Burckhardt**, University of Lausanne, Lausanne, Switzerland.

## Data Availability

The original contributions presented in the study are included in the article/Supplementary Material, further inquiries can be directed to the corresponding author.
