# Supplementary material for: Combined Vitamin D, Omega-3 Fatty Acids, and a Simple Home Exercise Program May Reduce Cancer Risk Among Active Adults Aged 70 and Older: A Randomized Clinical Trial
Source: Front Aging. 2022 Apr 25;3:852643. doi: 10.3389/fragi.2022.852643 (PMC9261319; doi:10.3389/fragi.2022.852643)
Supplement: Supplementary file 1 [file DataSheet1.docx]

**Supplement**

**Combined vitamin D, omega-3 fatty acids and a simple home exercise program may reduce cancer risk among active adults age 70 and older: a randomized clinical trial**

Heike A. Bischoff-Ferrari; Walter C. Willett; JoAnn E. Manson; Bess Dawson-Hughes; Markus G. Manz; Robert Theiler; Kilian Brändle; Bruno Vellas; René Rizzoli; Reto W. Kressig; Hannes B. Staehelin; José A. P. da Silva, Gabriele Armbrecht; Andreas Egli; John A. Kanis; Endel J. Orav; Stephanie Gaengler; for the DO-HEALTH Research Group.

Contents

[Table S 1 Description of the home-based physical exercise program and the control exercise 2](#_Toc92703263)

[Table S 2 P-values for effect modification by subgroups 2](#_Toc92703264)

[Figure S 1 Sensitivity Analysis – reported cases of invasive cancer 3](#_Toc92703265)

[Figure S 2 Effect of treatments on cancer mortality 3](#_Toc92703266)

[eAppendix 1. Data sharing statement 4](#_Toc92703267)

### Table S 1 Description of the home-based physical exercise program and the control exercise

| **SHEP (Strength exercise)** | **Control exercise (Flexibility exercise)** |
| --- | --- |
| 1. Sit-to-stand (quadriceps /hip extension strength training)  2. One-leg stance (hip muscles strength training plus static balance training)  3. Pull Backs against elastic resistance (seated position)  4. External shoulder rotation against elastic resistance (seated position)  5. Steps | 1. Hip and knee mobility (seated position)  2. Hip mobility (standing position)  3. Trunk and chest mobility (seated position)  4. Shoulder mobility (seated position)  5. Ankle mobility (standing position) |

### Table S 2 P-values for effect modification by subgroups

| Subgroup variable | Treatment  group | Vitamin D | Omega-3s | SHEP |
| --- | --- | --- | --- | --- |
| Female | 0·191 | 0·453 | 0·498 | 0·110 |
| Age 75 | 0·340 | 0·608 | 0·434 | 0·518 |
| Low 25(OH)D at baseline | 0·656 | 0·331 | 0·310 | 0·975 |
| High omega-3 PUFAs at baseline | 0·973 | 0·296 | 0·284 | 0·085 |
| Overweight (BMI>25) | 0·423 | 0·402 | 0·690 | 0·626 |
| Obese (BMI>30) | 0·629 | 0·532 | 0·974 | 0·713 |

### Figure S 1 Sensitivity Analysis – reported cases of invasive cancer


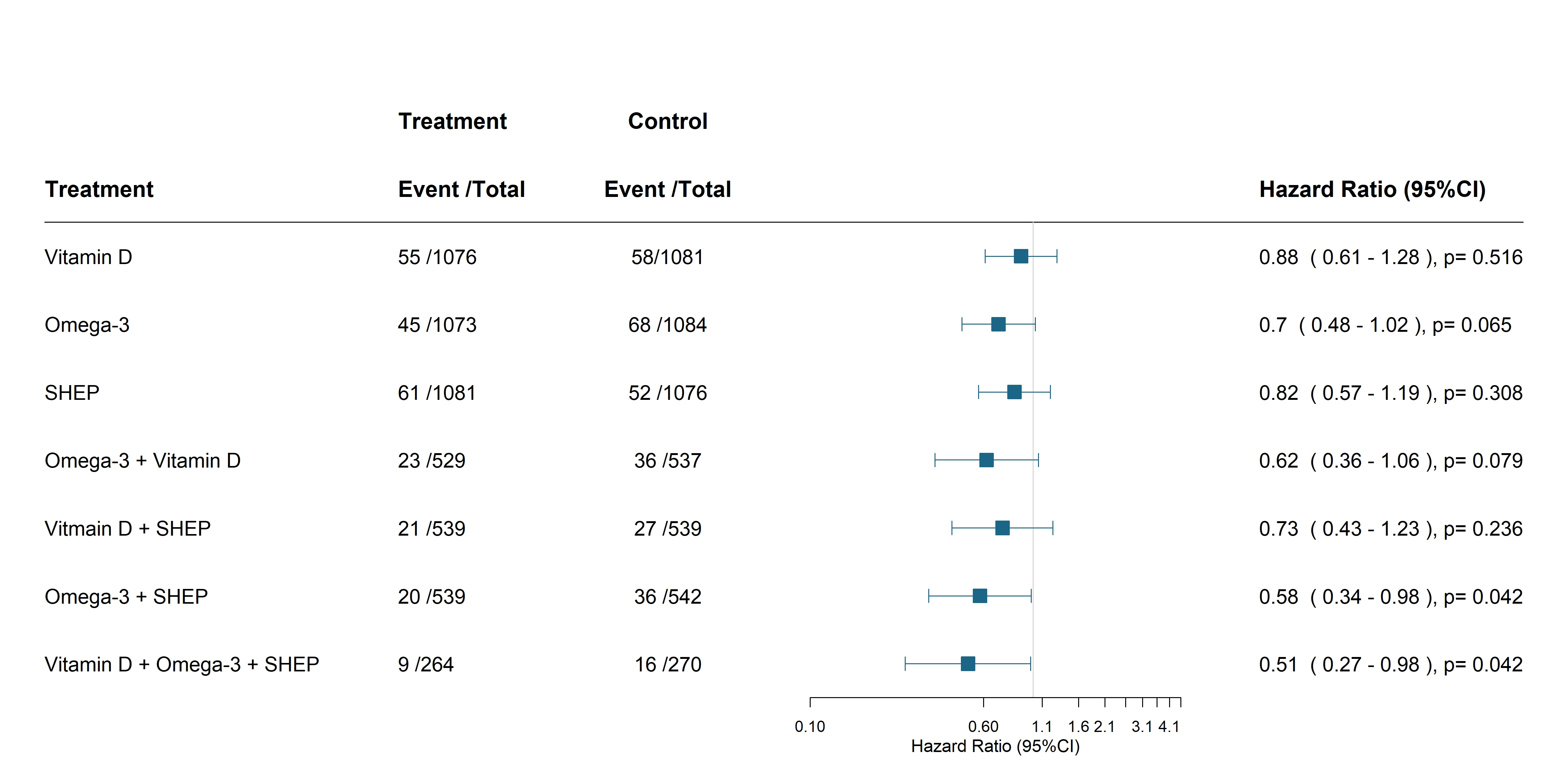


Legend Figure S1: Cox-proportional hazards model adjusted for history of cancer, sex, BMI, prior fall, age, and study centre. The comparison group is always the group that does not have the respective treatment(s) of interested. For all three treatments, it is the group who received only placebo. All reported invasive cancer cases minus 6 verified non-cases (n = 113) among 2157 participants.(Abbreviation: SHEP- Simple home exercise program)

### Figure S 2 Effect of treatments on cancer mortality

**
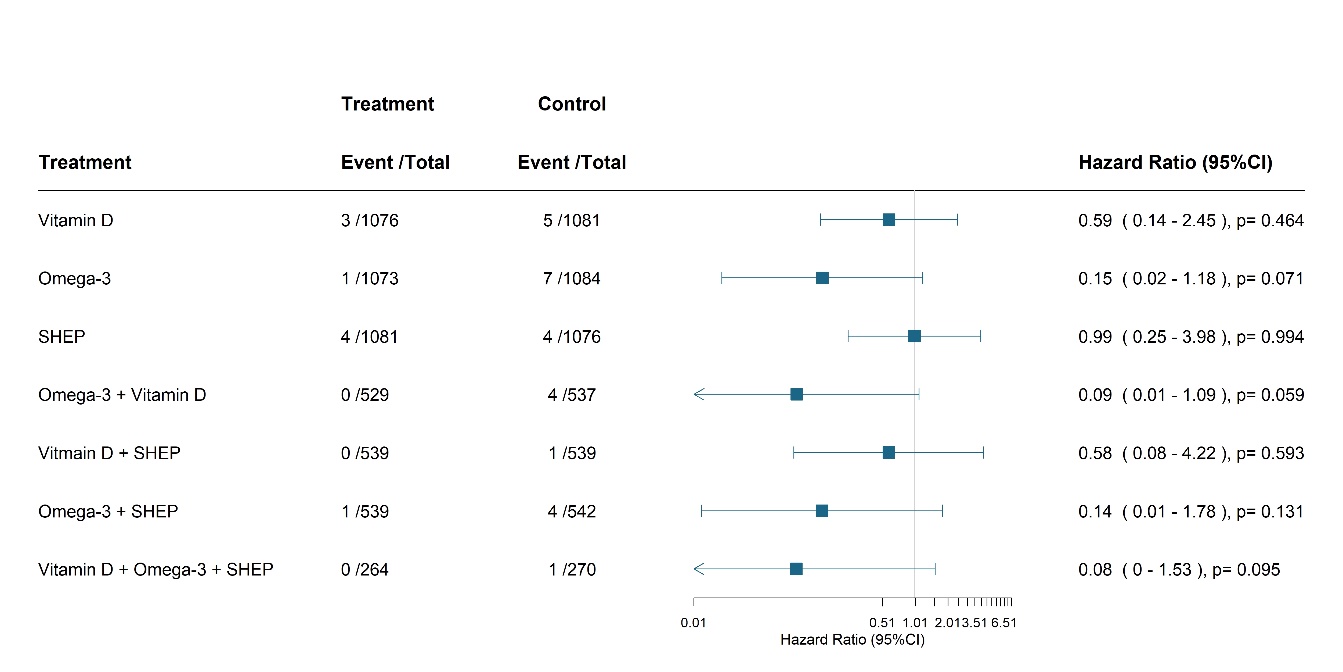
**

Legend Figure S2: There were only 8 cancer mortality cases over the 3-year duration of DO-HEALTH. Cox-proportional models are unadjusted due to the small cancer death numbers (n=8). (Abbreviation: SHEP- Simple home exercise program)

### eAppendix 1. Data sharing statement

Data Sharing statement: In a first step, no data will be made available to researchers external to DO-HEALTH Research Group to allow primary researchers to fully exploit the dataset. The data will be shared in a second step according to a controlled access system.
